# Insights into the Activity Change of Spore Photoproduct Lyase Induced by Mutations at a Peripheral Glycine Residue

**DOI:** 10.3389/fchem.2017.00014

**Published:** 2017-03-28

**Authors:** Linlin Yang, Lei Li

**Affiliations:** ^1^Department of Chemistry and Chemical Biology, Indiana University-Purdue University IndianapolisIndianapolis, IN, USA; ^2^Department of Dermatology, Indiana University School of MedicineIndianapolis, IN, USA

**Keywords:** radical transfer, spore photoproduct lyase, mutation, protein structure, radical SAM enzymes

## Abstract

UV radiation triggers the formation of 5-thyminyl-5,6-dihydrothymine, i.e., the spore photoproduct (SP), in the genomic DNA of bacterial endospores. These SPs, if not repaired in time, may lead to genome instability and cell death. SP is mainly repaired by spore photoproduct lyase (SPL) during spore outgrowth via an unprecedented protein-harbored radical transfer pathway that is composed of at least a cysteine and two tyrosine residues. This mechanism is consistent with the recently solved SPL structure that shows all three residues are located in proximity and thus able to participate in the radical transfer process during the enzyme catalysis. In contrast, an earlier *in vivo* mutational study identified a glycine to arginine mutation at the position 168 on the *B. subtilis* SPL that is >15 Å away from the enzyme active site. This mutation appears to abolish the enzyme activity because endospores carrying this mutant were sensitive to UV light. To understand the molecular basis for this rendered enzyme activity, we constructed two SPL mutations G168A and G168R, examined their repair of dinucleotide SP TpT, and found that both mutants exhibit reduced enzyme activity. Comparing with the wildtype (WT) SPL enzyme, the G168A mutant slows down the SP TpT repair by 3~4-fold while the G168R mutant by ~ 80-fold. Both mutants exhibit a smaller apparent (^D^*V*) kinetic isotope effect (KIE) but a bigger competitive (^D^*V/K*) KIE than that by the WT SPL. Moreover, the G168R mutant also produces a large portion of the abortive repair product TpT-${\text{SO}}_{2}^{-}$; the formation of which indicates that cysteine 141 is no longer well positioned as the H-donor to the thymine allylic radical intermediate. All these data imply that the mutation at the remote glycine 168 residue alters the enzyme 3D structure, subsequently reducing the SPL activity by changing the positions of the essential amino acids involved in the radical transfer process.

## Introduction

**Figure 1:**
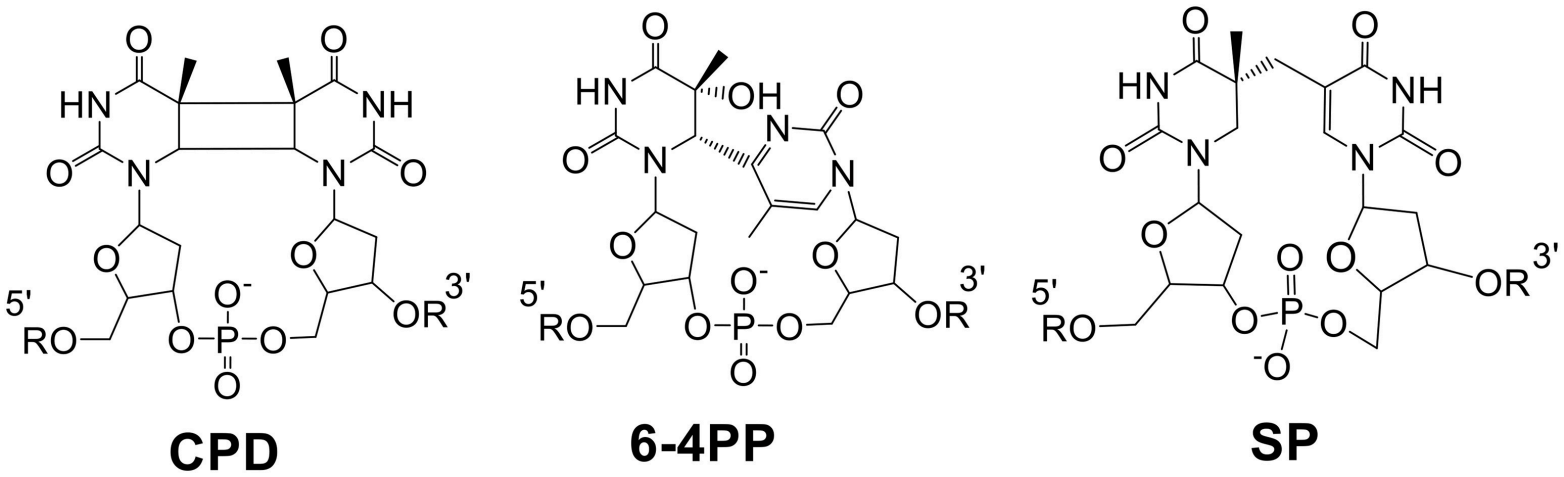
**Chemical structures of the three thymine dimers, all of which can be repaired via direct reversal mechanisms**. CPDs and 6-4PPs are repaired by light-dependent photolyases and SP is repaired by SPL which is light-independent but energy-dependent.

DNA photoreaction induced by the UV portion of solar light is one of the most universal reactions occurring on our planet. The resulting DNA photodamages, if not repaired in time, may lead to genome instability and potential cell death. Among the four deoxyribonucleotides, thymidine is the most UV sensitive base and UV radiation of which normally promotes its dimerization with a neighboring pyrimidine residue. Such a dimerization reaction in typical vegetative cells leads to the formation of cyclobutane pyrimidine dimers (CPDs) and pyrimidine (6-4) pyrimidone photoproducts (6-4PPs) (Figure 1). In contrast, 5-thyminyl-5,6-dihydrothymine that is commonly referred to as the spore photoproduct (SP) is the dominant DNA photolesion found in bacterial endospores.

All three dimers can be repaired via a direct reversal mechanism, i.e., the dimer is reverted back to two pyrimidine residues by breaking the crosslinking bonds. Direct reversal repair of CPDs and 6-4PPs is performed by specific photolyases (Sancar, 2003, 2008; Li et al., 2010; Liu et al., 2011; Benjdia, 2012; Kneuttinger et al., 2014), which use light and flavin cofactors to generate radical species in the dimers before bond scission occurs resulting in two pyrimidine residues. In contrast, the repair of SP in germinating spores is conducted mainly by a so-called radical SAM (*S*-adenosylmethionine) enzyme—the spore photoproduct lyase (SPL) (Sofia et al., 2001; Frey and Magnusson, 2003; Li, 2012; Yang and Li, 2013, 2015; Broderick et al., 2014). SPL initiates SP repair by reductively cleaving the SAM cofactor to yield a 5′-deoxyadenosyl (5′-dA) radical, which subsequently catalyzes the SP reversal process via a cascade of radical transfer processes. Protein sequence analysis has identified a homologous region in the carboxyl-terminal portions of the CPD photolyases and SPL, indicating that these enzymes may have descended from a common ancestral protein (Fajardo-Cavazos et al., 1993).

**Figure 2:**
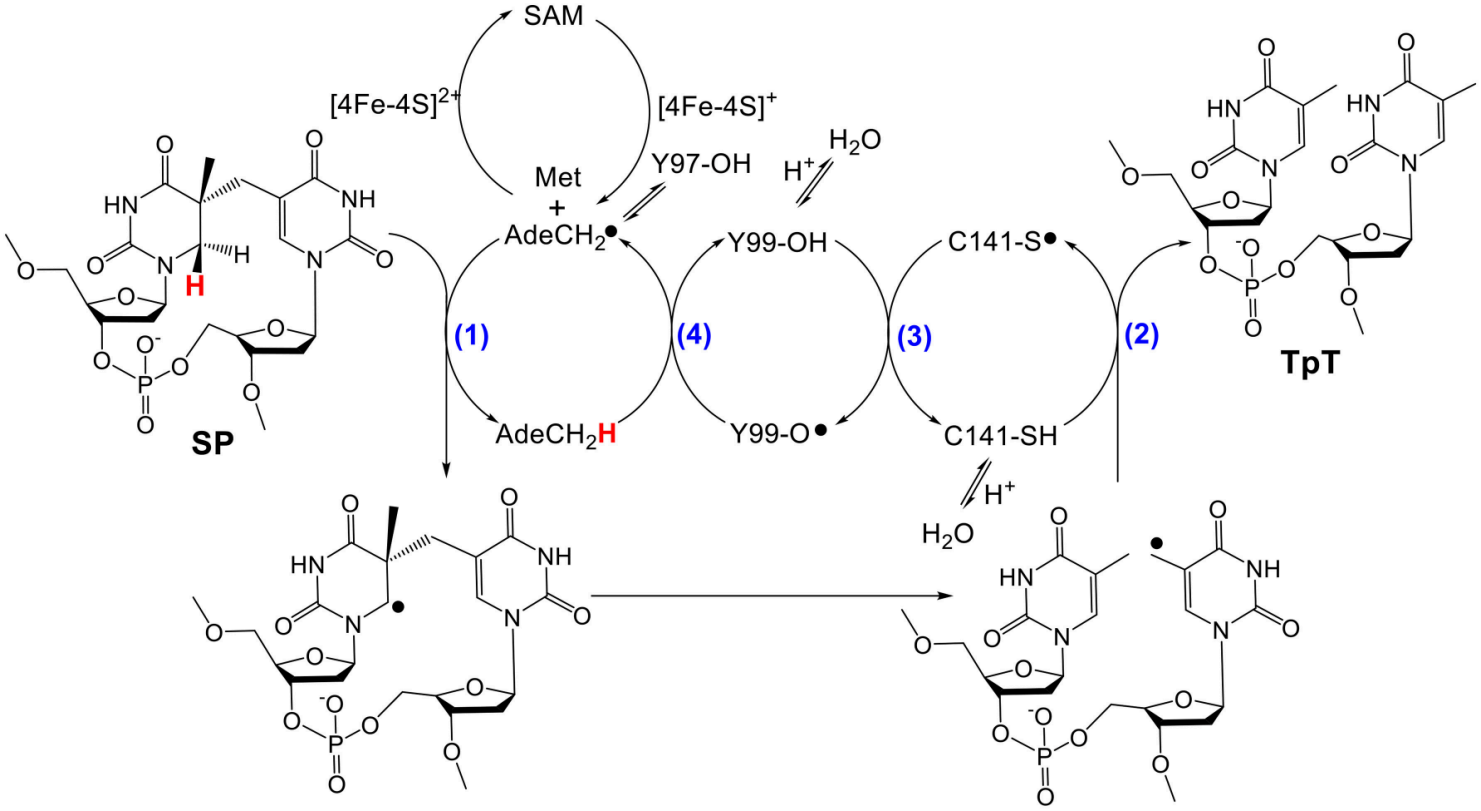
**Currently hypothesized reaction mechanism for SPL [amino acid residues are numbered according to the protein sequence in ***Bacillus subtilis*** (***Bs***) SPL]**. This mechanism implies that SPL uses a minimum of four H-atom transfer processes (numbers in blue) in each catalytic cycle. Y97 is hypothesized to facilitate the H-abstraction step from the methyl group of 5′-dA by delocalizing the radical at the resulting 5′-dA•.

As shown in Figure 2, the current mechanism implies that SPL harbors an unprecedented radical transfer pathway that is composed by at least one cysteine and two tyrosine residues (Donnellan and Stafford, 1968; Wang and Rupert, 1977; Setlow and Li, 2015). During the SPL-catalyzed SP repair process, after the resulting 5′-dA radical abstracts the H_6*proR*_ atom at SP (Mehl and Begley, 1999; Yang et al., 2011), the resulting thymine allylic radical accepts an H-atom from the conserved cysteine (C141 in *B. subtilis* (*Bs*) SPL), to produce a thiol radical and two repaired thymine residues (Fajardo-Cavazos et al., 2005; Yang et al., 2012). The thiol radical takes an H-atom from the neighboring Y99_(*Bs*)_; the Y99_(*Bs*)_ radical then oxidizes 5′-dA with the assistance of Y97, regenerating the 5′-dA radical before regenerating the SAM molecule (Yang et al., 2013). This reaction mechanism is supported by a recently solved SPL structure (Benjdia et al., 2012), which shows that the conserved cysteine and tyrosine residues are located in the SP binding pocket with distances among them feasible to support the hypothesized H-abstraction steps.

To help reveal the mechanism of SPL-mediated repair process, Fajardo-Cavazos et al. performed a number of mutations on the *spl-1* gene and identified a G168R_(*Bs*)_ mutant in a previously reported study (Fajardo-Cavazos and Nicholson, 1995). *B. subtilis* endospores carrying this mutant were sensitive toward UV radiation, indicating that the G→R mutation may inactivate the SPL enzyme (Fajardo-Cavazos and Nicholson, 1995). Although, the molecular mechanism for such an enzyme inactivation was unclear by then, it was reasonable to assume that the glycine replacement by a much larger and positively charged arginine may drastically alter the protein 3D structure, abolishing its SP repair function.

**Figure 3:**
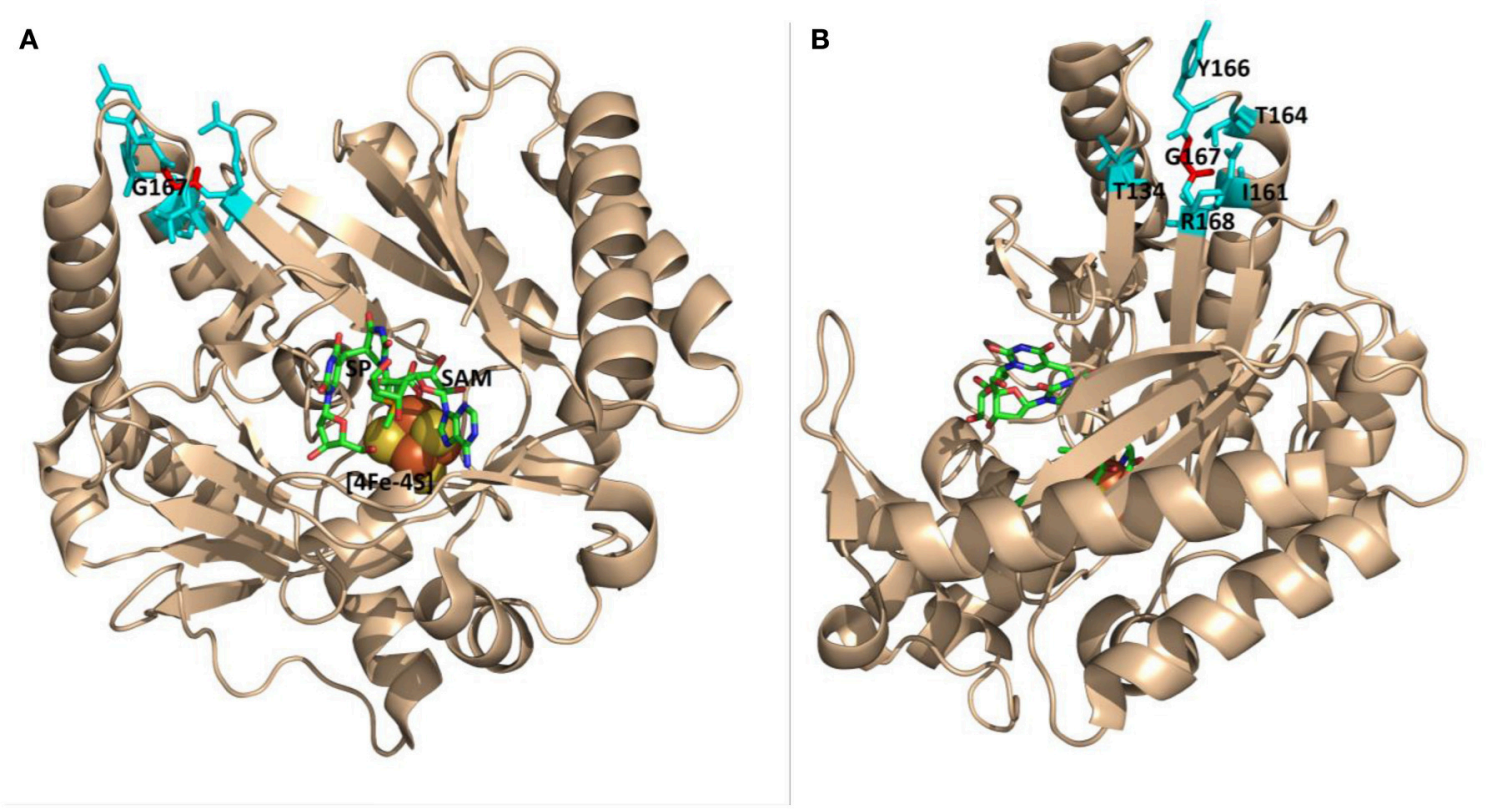
**The ribbon structure of ***Geobacillus thermodenitrificans*** (***Gt***) SPL in complex with SP and SAM viewed from different angles. (A)** The G167 _(*Gt*)_ residue (labeled in red) corresponds to the G168_(*Bs*)_ residue. It is located at the junction of a β-strand and a peptide loop, which appears flexible enough to accommodate various amino acid mutations. This residue is >16 Å from the bound SP molecule. **(B)** The structure viewed from a different angle that shows amino acid residues surrounding G167 (PDB code 4FHD, Benjdia et al., 2012).

Recently, the crystal structure of SPL from the bacterium *Geobacillus thermodenitrificans* (*Gt)* was solved (Benjdia et al., 2012), providing the structural basis for the analysis of the enzyme activity altered by amino acid mutations. An examination of the SPL_(*Gt*)_ structure indicates that G168_(*Bs*)_ (corresponding to G167_(*Gt*)_ residue) is located at the far end of a β-strand in connection with a flexible peptide loop (Figure 3B); the β-strand points to the SAM molecule and is ~16 Å away from the SP molecule bound (Figure 3). Although, an arginine is much bigger than a glycine and carries a positive charge at its guanidinium moiety under physiological pH, the seemingly flexible environment around the glycine may readily accommodate such a size increase without causing much enzyme activity change. Thus, the inactivation of the SPL enzyme by this G→R mutation is somewhat surprising.

To understand how the G168R mutation alters the enzyme function, we expressed the SPL G168R mutant in *E. coli* and examined its *in vitro* activity using dinucleotide SP TpT as a substrate. Our results confirmed that the G168R mutation drastically slows down the SP repair process (~80-fold) as expected. Further, we have mutated G168 to a similarly-sized alanine residue and examined the SPL G168A activity. Our data indicate that even this G→A mutation reduces the SPL activity by roughly 4-fold. Moreover, using deuterated dinucleotide SP TpT as the substrate, we found that the kinetic isotope effects are changed in similar trends for both G168A and G168R mutants comparing with those observed in the wildtype (WT) enzyme, indicating that these mutations alter the protein function under a similar mechanism. Moreover, the G168R mutant generates a large amount of TpT-${\text{SO}}_{2}^{-}$ that is formed after the thymine allylic radical intermediate is quenched by the externally added dithionite anion (Chandor-Proust et al., 2008; Yang et al., 2012; Benjdia et al., 2014), indicating that the position of the essential cysteine that serves as the H-donor to the thymine allylic radical intermediate is changed. Taken together, our results highlight the impacts of amino acid substitution at a seemingly insignificant position that however alters the protein structure and the catalytic function. The impacts are likely unique to SPL because it relies on the precise positioning of the essential amino acids for efficient radical transfer and enzyme catalysis.

## Methods

### Materials

DNA-modifying enzymes were purchased from Fermentas Life Sciences (Glen Burnie, MD). Oligonucleotide primers were synthesized by Integrated DNA Technologies (Coralville, IA). *E. coli* BL21 (DE3) and the expression vector pET-28a were purchased from Novagen (Madison, WI). All other buffers and chemicals were of the highest grade commercially available from Fisher, VWR or Sigma-Aldrich.

UV−visible spectra were recorded using UV-Mini 1240 Spectrophotometer and the associated data manager software package. The photoreaction was carried out using a Spectroline germicidal UV lamp (Dual-tube, 15 w, intensity: 1,550 uw/cm^2^) with samples ~5 cm from the lamp. The protein purification and the enzyme reactions were carried out under an inert atmosphere using a Coy lab anaerobic chamber (Grass Lake, MI) with the H_2_ concentration around 3%. DNA sequencing was performed by the GENEWIZ Inc. at South Plainfield, NJ.

### Preparation of SP substrates

The dinucleotide SP TpT was synthesized as previously described (Lin and Li, 2010). The *d*_4_-SP was photochemically synthesized using dinucleotide *d*_4_-TpT containing a–CD_3_ moiety at the 3′-thymine in a dry film reaction (Lin and Li, 2010).

### Construction of the SPL G168R/A expression vector

The *splB* gene was cloned from the *B. subtilis* chromosomal DNA (strain 168) into the pET-28a vector with a N-terminal His_6_-tag as described previously (Yang et al., 2011). Synthetic oligonucleotide primers 5′-GATCTCGCAAAGCTTCGATTTGTAACGAAATTTC-3′/5′-CAAATCGAAGCTTTGCGAGATCACTTTGGCC-3′ were used to generate the G168A mutant, and 5′-GATCTCCGAAAGCTTCGATTTGTAACGAAATTTC-3′/5′-CAAATCGAAGCTTTCGGAGATCACTTTGGCC-3′ were employed to generate the G168R mutant. Both mutations were conducted using the Stratagene QuikChange site-directed mutagenesis kit following the manufacturer's instruction.

### Expression and purification of SPL mutants

Both the SPL G168A and G168R mutants were expressed in LB medium containing appropriate antibiotics as previously described (Yang et al., 2011). The proteins were purified via Ni-NTA chromatography. The bound protein was washed using a buffer containing 25 mM Tris, 250 mM NaCl, 20 mM imidazole, and 10% glycerol (pH 7.0) for 10 column volumes. The protein was then eluted by the same buffer containing 250 mM imidazole. The resulting protein was dialyzed against the same buffer without imidazole, aliquoted to 150 mL Eppendorf tubes, flash frozen in liquid N_2_ and stored in −80°C freezer. Our previous research showed that the SPL protein may contain some contaminating enzyme that can degrade the 5′-dA generated from the SP repair reaction (Yang et al., 2012); no contaminating proteins were found here as indicated by the high stability of 5′-dA.

For protein samples used for circular dichroism spectrometric analysis, the samples were further purified by ion exchange chromatography using the SP Sepharose fast flow ion exchange resin (GE Healthcare Life Sciences, Piscataway, NJ). The protein was then eluted using the same buffer containing 450 mM NaCl instead. The resulting protein was diluted by 3-fold to reduce the salt concentration to 150 mM. Judged by SDS-PAGE, the proteins after the ion-exchange chromatography were >97% pure.

### Protein, iron, and sulfide assays

Protein concentrations were determined by the method of Bradford (Bradford, 1976). Iron content was determined using *o*-bathophenanthroline (OBP) as described by Fish (1988) and sulfide assays were carried out using the method described by Beinert (1983). The detailed assay description can be found in our previous publications (Yang et al., 2011, 2012, 2017).

### [4Fe-4S] cluster reconstitution

The [4Fe-4S] cluster reconstitution was conducted inside the Coy chamber at ambient temperature (22°C) as previously described (Yang et al., 2011).

### SPL activity assay

SPL activity was analyzed as previously described (Yang et al., 2013). Typically, a reaction mixture contained 2 nmole SPL, 20 nmole SP TpT, and 20 nmole SAM in 400 μL buffer containing 25 mM Tris-HCl, 250 mM NaCl and 10% glycerol at pH 7.0. These components were incubated for 30 min under an inert atmosphere at ambient temperature to allow SPL enough time to find its substrate before 200 nmole sodium dithionite was added to reduce the [4Fe-4S] cluster and initiate the enzyme reaction. The reaction was carried out under anaerobic conditions at ambient temperature (22°C) for various periods of time. At each time point, 40 μL of the solution was aliquoted to an Eppendorf tube containing 2 μL 3M HCl as described in our previous studies (Yang et al., 2011, 2012, 2013). After removing protein via centrifugation, the supernatants were loaded onto HPLC, separated, and analyzed using procedures described below.

### Deuterium kinetic isotope effects (KIEs)

The apparent (^D^*V*) KIEs for the G168A_(__*Bs*__)_ and G168R_(__*Bs*__)_ mutants were measured by direct comparison of the initial rates with 1 mM of SP TpT and *d*_4_-SP TpT as the enzyme substrate respectively (Yang et al., 2011, 2012). The competitive [^D^(*V/K*)] KIE was determined via the internal competition approach using an equimolar mixture of SP TpT and *d*_4_-SP TpT (1 mM total concentration). In our experiments, the KIEs at varying time points were measured at relatively low extents of reaction of between 1 and 15%, where the isotopic composition of SPs varies approximately linearly with the extent of reaction (Kohen and Limbach, 2006). The competitive KIE, therefore, was calculated by linear extrapolation of the KIEs measured by the LC/MS assay described below to zero extent of reaction when the concentrations of SP TpT and *d*_4_-SP TpT were equal (Li and Marsh, 2006; Yang et al., 2011, 2013).

### HPLC assay and reaction rate determination

HPLC was performed with detection at 260 nm using a Shimadzu LC-20AB high-pressure gradient solvent delivery unit coupled with a SPD-20A UV-vis detector and a Waters XBridge™ OST reverse-phase C18 column (2.5 μm, 4.6 × 50 mm). The analysis of SP TpT repair was conducted using our previously described HPLC procedure where 10 mM triethylammonium acetate (TEAA), pH 7.0, was used as Mobile Phase A and compounds were eluted with an ascending gradient (0–25%) of Mobile Phase B which was composed of 67% Mobile Phase A and 33% acetonitrile at a flow rate of 1 mL/min (Yang et al., 2011, 2012; Yang and Li, 2013). The resulting 5′-dA, TpT, and TpT${\text{SO}}_{2}^{-}$ peaks in the HPLC chromatograms were integrated and the integrations plotted against reaction time. Using the HPLC calibration curve generated with authentic 5′-dA and TpT (Yang et al., 2011) as well as TpT${\text{SO}}_{2}^{-}$ (Yang et al., 2012), the formation rates of these species were calculated. All experiments were repeated for at least three times and the average from these determinations was reported. The maximum deviation of these determinations from the average defines the error range.

### LC-MRM-MS analysis

SP repair by SPL was analyzed via an Agilent 1200–6410 LC-MS triple quadrupole mass spectrometer operating in the multiple reaction monitoring (MRM) mode. As revealed in previous studies (Ciccimaro and Blair, 2010; Liu et al., 2014; Wagner et al., 2015), linear responses between the intensity of mass spec signals and the amount of the analytes were observed, demonstrating the feasibility of using this LC-MRM-MS assay for quantitative analysis. The HPLC experiments used a ZORBAX Eclipse plus C18 column 4.6 × 50 mm (3.5 μm in particle size, Agilent Technologies, Santa Clara, CA) at ambient temperature, following the experimental procedures described in our previous studies (Yang et al., 2017).

### CD spectropolarimetry

To explore the possible protein conformational changes induced by a mutation at G168, we used CD spectropolarimetry under a protocol adopted by the McLeish laboratory (Najbar et al., 1997, 2000). CD spectra were recorded on a Jasco J-810 spectropolarimeter. The instrument was calibrated using D-10-camphorsulfonic acid. Quartz cells (Hellma) having a path length of 0.1 cm were employed and were maintained at 25°C using a Neslab RTE-110 circulating water bath. Spectra were recorded in the 200−260 nm wavelength range with 0.5 nm increments and 1 s integration time. Spectra were an average of three scans recorded at a scan speed of 50 nm/min. SPL protein concentrations were between 5 and 15 μM as determined by UV-vis analysis at 280 nm (Greenfield, 2006). Proteins are in a buffer containing 25 mM Tris, 250 mM NaCl, and 10% glycerol at pH 8. A spectrum with only the buffer provided the blank.

## Results

### Protein expression and purification

**Figure 4:**
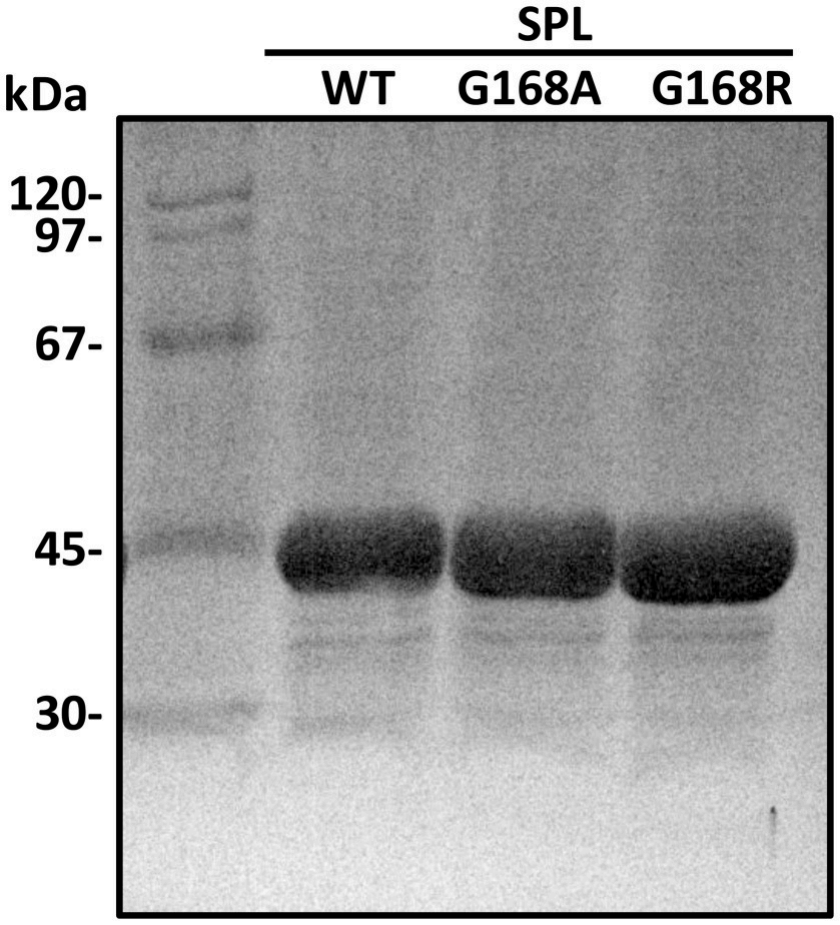
**The SDS-PAGE gel of the SPL proteins overexpressed in ***E. coli*** and purified via Ni-NTA chromatography**.

The protein expression level in *E. coli* is suggested to be similar for all three proteins, i.e., WT SPL, G168A and G168R, indicating that the mutations at the glycine do not alter the protein folding drastically enough to change the protein solubility. All three proteins were purified by Ni-NTA chromatography under a strictly inert atmosphere as dark-brown solutions indicating the presence of the iron-sulfur cluster. The purity of the proteins was checked by SDS-PAGE to be >95% and the purified SPL proteins exhibited a single dominant band at ~40 kDa (Figure 4). The typical yield for the purification process is 20~40 mg protein per liter of LB media. The proteins were further characterized by ESI-MS spectrometry after being retrieved from the anaerobic chamber and immediately injected into the ESI mass spectrometer (Figure S1). As shown in the Figure S2 in the supporting information, the observed protein masses agree well with the predicted values, further confirming the availability of these mutants. Moreover, the WT SPL, SPL G168A, and SPL G168R mutants exhibit indistinguishable CD spectra (Figure S3), indicating that the mutation of glycine to alanine and arginine at the position 168 does not markedly alter the protein secondary structures.

### [4Fe-4S] cluster in SPL mutants

**Figure 5:**
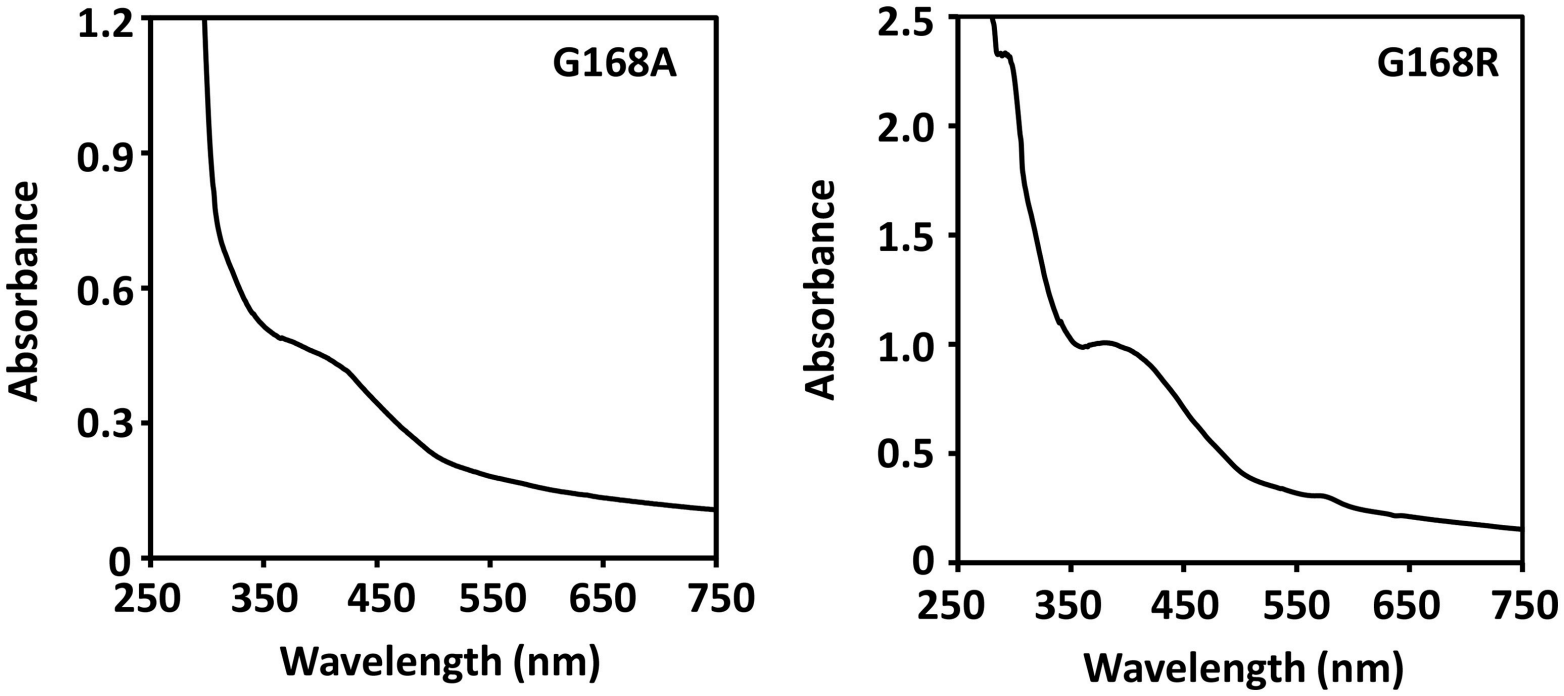
**UV-visible absorption spectra of the [4Fe-4S] cluster in the as-isolated SPL glycine mutants**. Both spectra exhibit a shoulder-like absorption around 420 nm that is the characteristic peak for a [4Fe-4S] cluster at the 2+ oxidation state. The spectra were taken in a 25 mM Tris buffer containing 250 mM NaCl, 10% glycerol, at pH 7.0.

The lack of drastic protein conformational changes after the glycine mutation suggests that the stability of the catalytically essential [4Fe-4S] cluster is unlikely altered as well. The as-isolated SPL mutants exhibited a typical UV absorption for a [4Fe-4S]^2+^ with a shoulder at 420 nm (Figure 5). Especially, the UV absorption spectrum exhibited by the G168R mutant confirms that the incorporation of the much bigger arginine residue does not alter the stability of the cluster that is essential for the enzyme activity in repairing SP. The presence of the cluster was also confirmed by the iron-sulfur content analysis. As expected, the as-isolated SPL G168A mutant contained 3.0 iron and 2.9 sulfur atoms per protein molecule; these numbers increased to 3.8 iron and 3.7 sulfur atoms after cluster re-constitution. Importantly, the as-isolated G168R mutant contained 2.9 iron and 2.8 sulfur atoms per molecule; both numbers increased to 3.5 upon cluster re-constitution. These numbers are comparable to the iron and sulfur contents found in WT SPL, that contains 3.1 iron and 2.9 sulfur atoms per protein in the as-isolated enzyme and 3.8 iron and 3.7 sulfur atoms after cluster reconstitution (Yang et al., 2011). The iron and sulfur contents further support the conclusion that the [4Fe-4S] cluster in both G168 mutants remains intact. The results are also consistent with the structural finding that glycine168 is distant from the [4Fe-4S] cluster; a mutation at this residue is unlikely to alter the cluster stability.

### SP TpT repair

**Figure 6:**
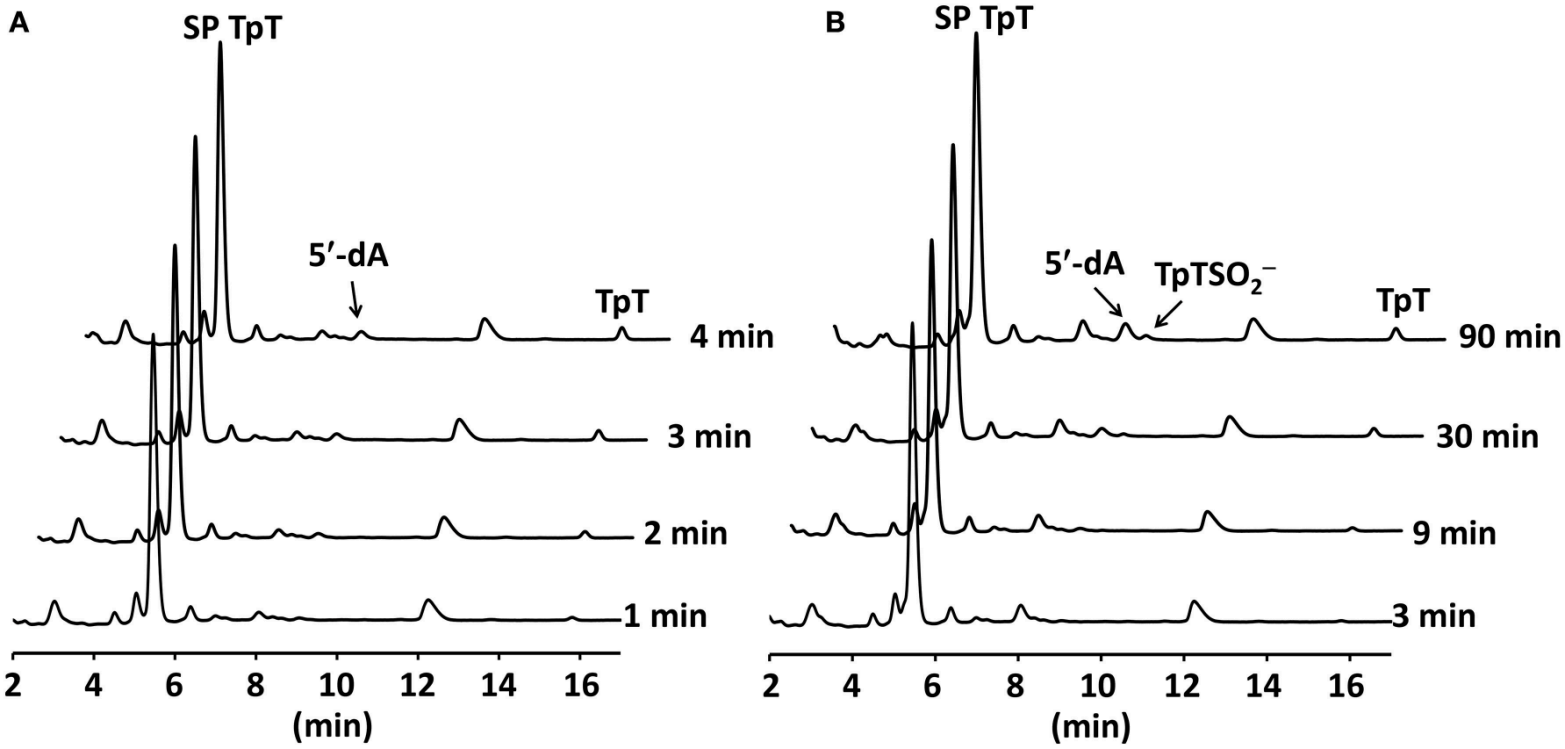
**(A)** HPLC chromatograms of SP TpT repair by the SPL G168A mutant. The repair generates dinucleotide TpT at ~0.09 min^−1^. **(B)** HPLC chromatograms of SP TpT repair by the SPL G168R mutant. The repair is at ~0.004 min^−1^; this is >20-fold slower than that by the G168A mutant. The reaction also produces a significant amount of TpT-${\text{SO}}_{2}^{-}$ that accounts for ~33% of all SP repaired.

The conservation of the [4Fe-4S] cluster in these mutant proteins indicates that these mutants may actively conduct the SP TpT repair. Considering that an alanine is only slight bigger than a glycine residue and position 168 is located at a flexible junction of a β-strand and a peptide loop, we expect that the SPL G168A mutant may exhibit a similar activity as the WT enzyme. A careful examination of the G168A mutant activity reveals that SP TpT was repaired to TpT at 0.09 ± 0.01 min^−1^ (Figure 6A), suggesting that the G→A mutation retains the enzyme activity. However, comparing with the repair efficiency of 0.35 ± 0.05 min^−1^ exhibited by the WT SPL (Yang et al., 2011, 2012, 2013), the activity of the G168A mutant is reduced by 3~4-fold.

Consistent with this trend, the replacement of this glycine by a much larger arginine residue further reduces the enzyme activity (Figure 6B). The repair rate was determined to be ~0.004 ± 0.001 min^−1^, which is roughly 20-fold lower than that of the G168A mutant and 90-fold lower than the WT enzyme. This drastically reduced enzyme activity is likely responsible for the high UV sensitivity of endospores carrying the G168R mutant observed previously (Fajardo-Cavazos and Nicholson, 1995). In addition to the reduced repair rate, the G168R mutant also produced a large portion of TpT-${\text{SO}}_{2}^{-}$ that accounts for one third of the SP TpT repaired. As shown in the previous studies (Chandor-Proust et al., 2008; Yang et al., 2012), TpT-SO2^−^ is the dominant SP TpT repair product by the SPL_(*Bs*)_ C141A mutant in which the intrinsic H-atom donor cysteine141 is no longer present and the vast majority of the thymine allylic radical intermediate is quenched by the externally added dithionite anion. A large portion of the intermediate is quenched by the dithionite in the G168R mutant reaction, implying that the position of cysteine141 must be changed in the G168R mutant protein.

### Apparent (^D^*V*) kinetics isotope effect (KIE) determination

To gain further insight into how these glycine mutants may impact the SPL activity, we compared the initial rates of the mutant reactions using SP TpT and *d*_4_-SP TpT as the substrates respectively. The *d*_4_-SP TpT contains a -CD_3_ group and a deuterium at the H_6*proR*_ position of the 5′-nucleoside (Lin and Li, 2010). The D_6*proR*_ atom abstraction step by the 5′-dA radical (Figure 1) is slow, that further slows the overall repair process, resulting in the ^D^*V*_*max*_ KIE in the steady state enzyme kinetics. Again, because the SPL G168A mutant possesses a very short “steady state,” we term the derived ^D^*V*_*max*_ KIEs as “apparent” (^D^*V*) KIEs, as shown in our previous SPL studies (Yang et al., 2011, 2012).

Our previous studies showed that the abstracted D_6*proR*_ is not returned to the repaired TpT (Yang et al., 2011); therefore only three deuterium atoms are retained in the TpT product. We found that the *d*_3_-TpT was produced at 0.037 ± 0.001 min^−1^ by the SPL G168A mutant. Comparing the rate with that from unlabeled SP leads to an apparent (^D^*V*) KIE of 2.6 ± 0.3. Similarly, the *d*_4_-SP TpT repair by the SPL G168R mutant was determined to be at 0.0017 ± 0.0003 min^−1^, leading to an apparent (^D^*V*) KIE of 2.5 ± 0.5, that is basically identical to that exhibited by the G168A mutant. However, both numbers are smaller than the (^D^*V*) KIE of 2.8 ± 0.3 found in the WT SPL_(*Bs*)_ reaction (Yang et al., 2011, 2013).

### Competitive (^D^*V/K*) KIE determination

**Figure 7:**
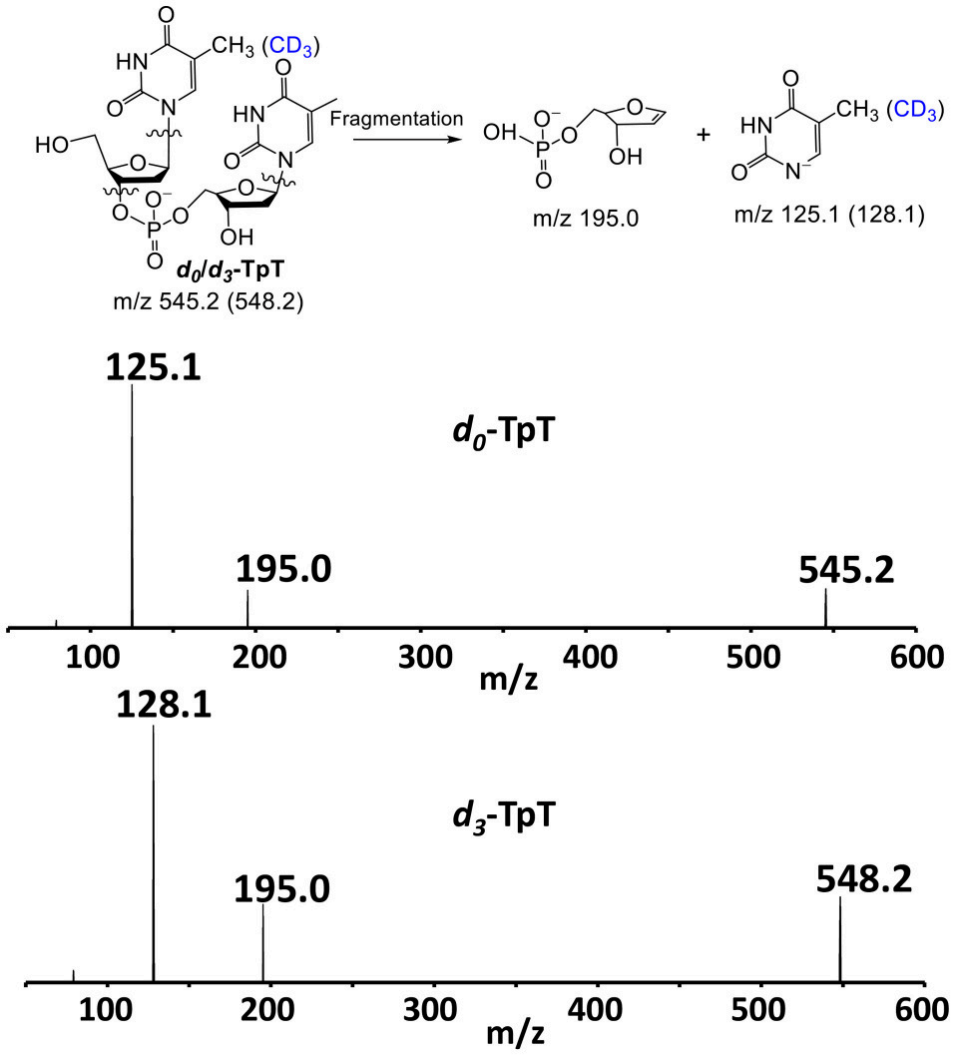
**The chemical structures of the repair products ***d***_**0**_-/***d***_**3**_-TpT and the two major fragments observed in the ESI-MS analysis under the negative ion mode**. The fragment signals of m/z 125.1 and 128.1 corresponding to the 5′-thymine daughter ion were used to quantify the ratio of the formed *d*_0_- and *d*_3_-TpT via MS/MS.

Besides the (^D^*V*) KIEs, we also measured the (^D^*V/K*) KIE for the SPL G168A reaction by using the 1:1 mixture of SP TpT and *d*_4_-SP TpT. The SP repair subsequently generates a mixture of TpT and *d*_3_-TpT; the relative amount of these two species was determined by the MS/MS analysis. As shown in Figure 7, one of the major product ions generated from the TpT fragmentation is the release of the 5′-thymine anion; the product ion from *d*_3_-TpT carries three deuterium atoms and thus exhibits a + 3 Da mass shift. Therefore, the fragment signal can be used as the marker to distinguish the unlabeled TpT from *d*_3_-TpT, enabling us to accurately measure the ratio between these two species.

**Figure 8:**
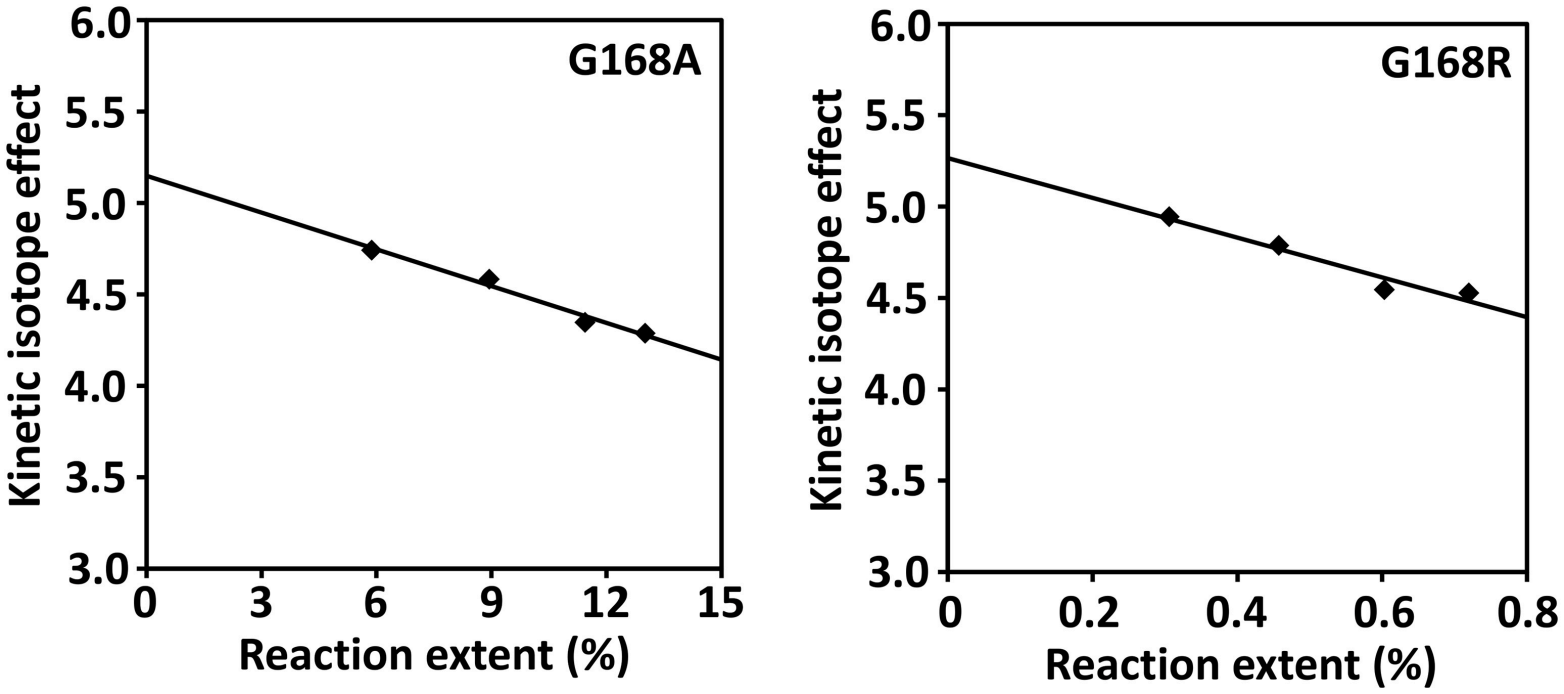
**Determination of the competitive (^**D**^***V/K***) isotope effect by extrapolation of the measured KIEs to zero extent of reaction for SPL G168A and G168R mutants**. The competitive KIE was determined to be 5.1 ± 0.5 for the G168A mutant and 5.3 ± 0.5 for the G168R mutant.

The competitive KIEs were measured at relatively low extents of reaction of <13%. Under these conditions the isotopic composition of the starting SP varies approximately linearly with the extent of reaction (Li and Marsh, 2006); the competitive (^D^*V/K*) KIE was calculated by linear extrapolation of the kinetic isotope effects measured at various reaction extents to zero extent reaction where the unlabeled SP and *d*_4_-SP are at 1:1 ratio (Figure 8). The extrapolation results in 5.1 ± 0.5 for the G168A mutant and 5.3 ± 0.5 for the G168R mutant at extent zero. To facilitate activity comparison, we also determined the competitive KIE for the WT SPL enzyme using our latest LC/MS assay and obtained a KIE of 4.6 ± 0.5. This number is slightly bigger than 3.4 ± 0.3 determined in our previous work (Yang et al., 2013).

## Discussion

### The glycine mutations do not alter SPL expression

SPs formed in dormant endospores have to be repaired during the first few hours of the spore germination and outgrowth process before the bacterial vegetative cells start gene transcription and protein synthesis (Moeller et al., 2007). Unrepaired SPs result in spore killing likely due to an overwhelmed SP repair system (Mason and Setlow, 1986). As an essential SP repair enzyme in endospores, it is critical for SPL to function effectively and efficiently. If its activity is too low to repair the vast majority of SPs during the short time window in spore germination and outgrowth, death appears to be inevitable for UV-irradiated endospores.

Therefore, the UV sensitivity exhibited by spores carrying the G168R mutation is likely induced by the reduced SPL activity (Fajardo-Cavazos and Nicholson, 1995). Since SPL is an iron-sulfur enzyme, it is important for one to check whether the mutation destabilizes the cluster. Comparison of the WT SPL with the G168A and G168R mutants however did not reveal a significantly altered expression level among these proteins. Moreover, both mutants exhibit similar CD spectra to that of the WT enzyme and they harbor an intact [4Fe-4S] cluster, indicating that the glycine mutations have little impact to the protein secondary structures and positions of the three conserved cysteines at the radical SAM motif. These cysteines are likely bound to the irons in a manner similar to that found in the WT enzyme. Such a conclusion is supported by the SPL_(*Gt*)_ crystal structure in which the glycine is found to be at least 18 Å away from the cluster (Benjdia et al., 2012).

### Enhanced formation of TpT-${\text{SO}}_{2}^{-}$ indicates that C141 has moved

Although, the protein secondary structures seem to be largely unchanged, the mutation at the glycine residue that is distant from the enzyme active site does induce subtle protein conformational changes that is responsible for the observed reduction of the enzyme activity. The result is particularly surprising for the G→A mutant as an alanine only possesses an extra methyl group at the side chain comparing with a glycine. To replace a G by an A residue is expected to generate negligible conformational changes especially when this residue is at the junction of a flexible β-strand and a peptide loop located at the peripheral region of the protein 3D structure. However, the fact that the G168A mutation reduces the SPL activity by 3~4-fold relative to the WT enzyme indicates this small size increase causes some subtle conformational changes that are sufficient to alter the SP repair process.

The same trend is further illustrated by the bigger G→R mutation that results in ~80-fold activity reduction relative to that exhibited by the WT enzyme. More interestingly, the G168R mutant produces a large portion of TpT-${\text{SO}}_{2}^{-}$ after the SP TpT repair, *such an observation provides the most direct evidence that the glycine mutation results in protein conformational changes*. As shown in the previous studies, TpT-${\text{SO}}_{2}^{-}$ was generated as the major product from the SP TpT repair by the C141A mutant in which the intrinsic H-donor to the thymine allylic radical, cysteine 141, is removed (Chandor-Proust et al., 2008; Yang et al., 2012). Additionally, TpT-${\text{SO}}_{2}^{-}$ was generated at ~20% yield (together with 80% TpT) during the repair of dinucleotide SP TpT by the SPL from the bacterium *Clostridium acetobutylicum* (*Ca*) (Yang et al., 2017). Different from the SPL_(*Bs*)_ in which the conserved cysteine (C141) and tyrosine (Y99) are located on the same side of the enzyme binding pocket, the conserved cysteine (C74) in SPL_(*Ca*)_ is projected to be at the opposite side to the conserved tyrosine, as indicated by the structural studies (Benjdia et al., 2012). In order to maintain the integrity of the radical transfer pathway shown in Figure 2, the pocket has to collapse to bring the two residues to proximity. Such a requirement determines that SPL_(*Ca*)_ possesses a very flexible substrate binding pocket. As a consequence, C74_(*Ca*)_ is less likely to be well-positioned as the H-donor, especially when dinucleotide SP TpT, the smallest substrate, is used (Yang et al., 2017). This subsequently suggests that the thymine allylic radical can readily dissociate from the enzyme and be quenched by the externally added sodium dithionite. Here, comparing with that found in SPL_(*Ca*)_, the SP TpT repair by the G168R mutant is even slower. In addition, the resulting TpT-${\text{SO}}_{2}^{-}$ accounts for ~33% of all SP TpT repaired by the G168R mutant, that is even higher than that by SPL_(*Ca*)_.

Taken together, our data indicate that the position of C141 must be altered moderately in the G168A mutant but relatively drastically in the G168R mutant. In the G168A mutant, C141 still functions as the H-atom donor to the thymine allylic radical although it probably no longer occupies the optimal position. In contrast, in the G168R mutant, C141 is pushed further away from the enzyme-bound SP, making it more difficult to donate the H-atom. The size increase at the glycine may push other amino acid residues into the enzyme active site to make it smaller and/or more rigid. Consequently, it may be difficult for the G168R mutant to adjust the position of C141 to restore its role as the H-donor via protein thermal motions, leading to a much slower enzyme reaction. Subsequently, more thymine allylic radicals are probably prematurely released and then quenched by the externally added sodium dithionite, resulting in more TpT-${\text{SO}}_{2}^{-}$ formation.

### Such a hypothesis is supported by the KIE results

Although, the G168A and G168R mutants lead to a different extent of enzyme deactivation, they exhibit almost the same (^D^*V*) and (^D^*V/K*) KIEs. This observation indicates that despite the various extent of structural disturbance to the protein, these two mutations likely alter the SPL activity via the same mechanism, i.e., inducing subtle protein conformational changes by pushing the C141 away from the SP substrate and/or potentially increasing the rigidity of the binding pocket. Such protein conformational changes also offer an explanation to the observed KIE changes in these two mutant proteins.

We use the method adopted by Northrop to analyze the KIE changes (Northrop, 1975) by defining three new parameters, *k*_a_, *k*_b_, and *k*_c_. Here, *k*_a_ is an apparent first-order rate constant for the breakdown of the enzyme-substrate complexes ES and ES' to free enzyme and substrate, *k*_b_ is an apparent first-order rate constant for the conversion of the first enzyme complex following substrate binding to the first enzyme complex immediately following the first irreversible step of the reaction, and *k*_c_ is the apparent first-order rate constant for the conversion of the enzyme complex immediately following the first irreversible step to free enzyme. Then, the (^D^*V*) and (^D^*V/K*) KIEs can be calculated by the two equations below (Northrop, 1975):

(1) $$\begin{array}{l} \frac{V_{\text{H}}}{V_{\text{D}}}=\frac{k_{\text{bH}}/k_{\text{bD}}+{\left(k_{\text{b}}/k_{\text{c}}\right)}_{\text{H}}}{{\left(k_{\text{b}}/k_{\text{c}}\right)}_{\text{H}}+1} \end{array}$$

(2) $$\begin{array}{l} \frac{{\left(V/K\right)}_{\text{H}}}{{\left(V/K\right)}_{\text{D}}}=\frac{k_{\text{bH}}/k_{\text{bD}}+{\left(k_{\text{b}}/k_{\text{a}}\right)}_{\text{H}}}{{\left(k_{\text{b}}/k_{\text{a}}\right)}_{\text{H}}+1} \end{array}$$

Although, *k*_b_ is smaller in both G168 mutants due to the slower quenching of the thymine allylic radical, we cannot predict how the (*k*_bH_/*k*_bD_) is altered in these mutants as both *k*_bH_ and *k*_bD_ are smaller. However, since this factor is included in both equations, its change induced by the glycine mutations should impact both the (^D^*V*) and (^D^*V/K*) KIEs. Comparing with the KIEs determined in the WT enzyme, opposite changes for the (^D^*V*) and (^D^*V/K*) KIEs were found in these glycine mutants. Our previous studies determined a primary apparent KIE of 2.9 ± 0.3 for SPL_(*Bs*)_(Yang et al., 2011, 2013), that is slightly bigger than the apparent KIEs of 2.6 ± 0.3 found for the G168A and 2.5 ± 0.3 for the G168R mutant. In contrast, the (^D^*V/K*) KIE was determined as 4.6 ± 0.3 for SPL_(*Bs*)_ using the same assay adopted in the current study, that is smaller than the (^D^*V/K*) KIE of 5.1 ± 0.5 for the G168A and 5.3 ± 0.5 for the G168R mutant. The opposite change in the (^D^*V*) and (^D^*V/K*) KIEs indicates that the changes of *k*_a_ and *k*_c_ from the WT SPL to the G168 mutants must be opposite suggesting that the glycine mutations disturb the binding of substrates and the release of products differently. On the other hand, given the error range in the determined KIEs, the possibility that the WT SPL and the two G168 mutants exhibit similar (^D^*V*) and (^D^*V/K*) KIEs cannot be excluded. Therefore, even if the glycine mutations indeed result in discrimination against the substrate or product comparing with the WT enzyme, the extent of discrimination is likely small.

Moreover, in addition to changes mainly induced by the primary KIEs, other possibilities such as changes in binding isotope effects (BIEs) and commitment factors may be also involved. BIEs are an inherent part of most isotope effect measurements but are usually assumed to be negligible although some studies have established surprisingly large BIEs with enzymes such as lactate dehydrogenase and thymidine phosphorylase (Schramm, 2007; Świderek and Paneth, 2013). It is certainly possible BIEs may play a role in KIE changes in the G168 mutants. Also, it is known that “forward commitment” and “reverse commitment” can make substrate binding and/or product release rate limiting and suppress KIEs (Schramm, 1998; Cleland, 2005) the changes in commitment factors may also contribute to the observed KIE changes here.

### Lessons learned from the glycine mutational studies

**Figure 9:**
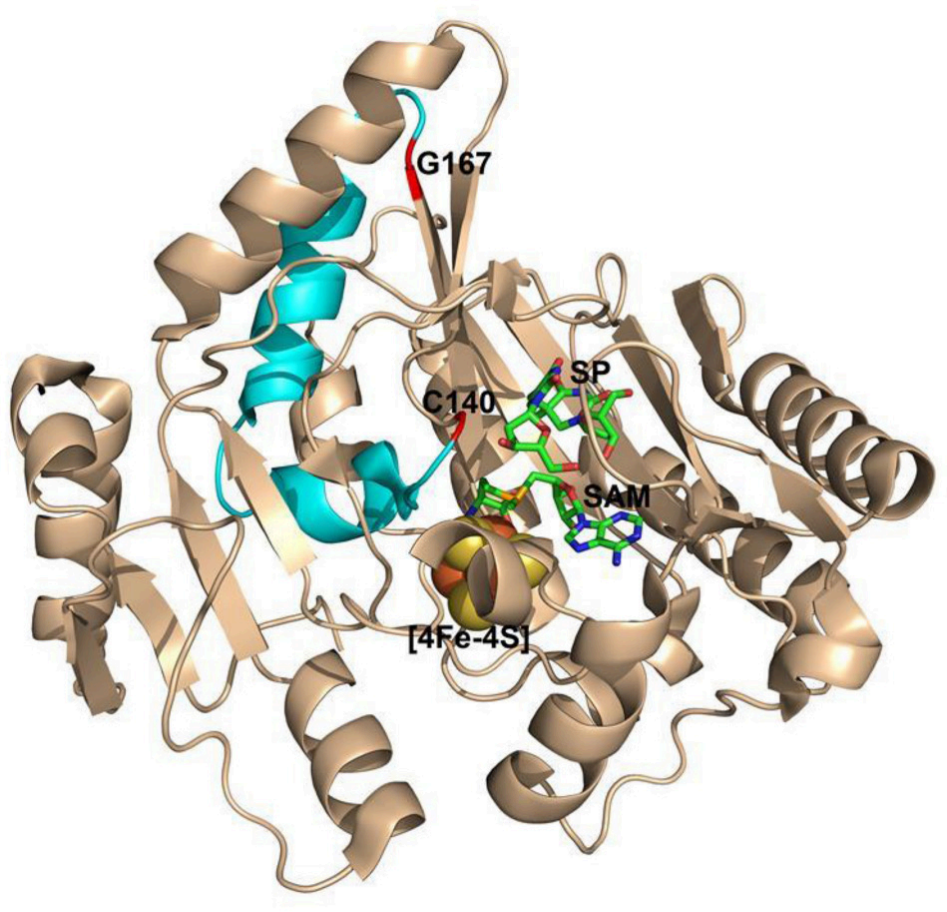
**The ribbon structure of ***Geobacillus thermodenitrificans*** (***Gt***) SPL in complex with SP and SAM**. The G167_(*Gt*)_ and C140_(__*Gt*__)_ residues are shown in red. They correspond to the G168_(*Bs*)_ and C141_(*Bs*)_ residues and are located at a loop-β-strand junction and a flexible loop respectively. They are separated by a helix-turn-helix motif colored in cyan and are found to be 17 Å away from each other. (PDB code 4FHD).

The results reported here are surprising because the glycine is located at a flexible region that is distant from the substrate binding pocket and the conserved cysteine141. Even so, the position of C141 is still changed as indicated by the TpT-${\text{SO}}_{2}^{-}$ formation in the G168R mutant. As shown in Figure 9, a helix-turn-helix motif is located between the two residues that are roughly 17 Å away from each other as measured from the SPL(_*Ca*__)_ structure (Benjdia et al., 2012). Given these residues are located at a loop and a loop-β-strand junction respectively, it is reasonable to assume that the size increase at the glycine may be well tolerated and the corresponding mutations may have little impact to the cysteine residue. However, even the smallest G→A mutation can reduce the enzyme activity by 3~4-fold, that may reflect the nature of the SPL enzyme that harbors a delicate radical transfer pathway. To maintain an efficient radical transfer process, the positions of the involving amino acid residues on the pathway must be strictly maintained. Even a small size change like the minimal G→A mutation at a remote site may induce subtle 3D conformational changes and drastically reduce the enzyme activity.

It is worth pointing out that our conclusion here is based on the repair of the dinucleotide SP TpT. In germinating spores, the SP lesions are embedded in the genomic DNA, in which the presence of nucleotides upstream and downstream to SP may result in stronger binding interactions with the enzyme. The resulting rigid DNA-enzyme framework may prevent the necessary protein conformational changes from occurring to enable the quenching of the thymine allylic radical, subsequently abolishing the SPL activity completely. But nevertheless, our conclusion, i.e., the size increase at a remote glycine changes the position of the essential cysteine and the overall substrate binding pocket and thus largely inactivates the enzyme, is still valid.

## Author contributions

LY performed the experiments. LL and LY wrote the manuscript.

### Conflict of interest statement

The authors declare that the research was conducted in the absence of any commercial or financial relationships that could be construed as a potential conflict of interest.

## References

[B1] Beinert H. (1983). Semi-micro methods for analysis of labile sulfide and of labile sulfide plus sulfane sulfur in unusually stable iron-sulfur proteins. Anal. Biochem. 131, 373–378. 10.1016/0003-2697(83)90186-0 6614472

[B2] Benjdia A. (2012). DNA photolyases and sp lyase: Structure and mechanism of light-dependent and independent DNA lyases. Curr. Opin. Struct. Biol. 22, 711–720. 10.1016/j.sbi.2012.10.002 23164663

[B3] Benjdia A. Heil K. Barends T. R. M. Carell T. Schlichting I. (2012). Structural insights into recognition and repair of UV-DNA damage by spore photoproduct lyase, a radical SAM enzyme. Nucleic Acids Res. 40, 9308–9318. 10.1093/nar/gks603 22761404 PMC3467042

[B4] Benjdia A. Heil K. Winkler A. Carell T. Schlichting I. (2014). Rescuing DNA repair activity by rewiring the H-atom transfer pathway in the radical SAM enzyme, spore photoproduct lyase. Chem. Commun. 50, 14201–14204. 10.1039/C4CC05158K 25285338

[B5] Bradford M. M. (1976). A rapid and sensitive method for the quantitation of microgram quantities of protein utilizing the principle of protein-dye binding. Anal. Biochem. 72, 248–254. 10.1016/0003-2697(76)90527-3 942051

[B6] Broderick J. B. Duffus B. R. Duschene K. S. Shepard E. M. (2014). Radical S-adenosylmethionine enzymes. Chem. Rev. 114, 4229–4317. 10.1021/cr4004709 24476342 PMC4002137

[B7] Chandor-Proust A. Berteau O. Douki T. Gasparutto D. Ollagnier-De-Choudens S. Fontecave M.. (2008). DNA repair and free radicals, new insights into the mechanism of spore photoproduct lyase revealed by single amino acid substitution. J. Biol. Chem. 283, 36361–36368. 10.1074/jbc.M806503200 18957420 PMC2662300

[B8] Ciccimaro E. Blair I. A. (2010). Stable-isotope dilution LC-MS for quantitative biomarker analysis. Bioanalysis 2, 311–341. 10.4155/bio.09.185 20352077 PMC2843934

[B9] Cleland W. W. (2005). The use of isotope effects to determine enzyme mechanisms. Arch. Biochem. Biophys. 433, 2–12. 10.1016/j.abb.2004.08.027 15581561

[B10] Donnellan J. E. Jr Stafford R. S. (1968). The ultraviolet photochemistry and photobiology of vegetative cells and spores of *Bacillus megaterium*. Biophys. J. 8, 17–28. 10.1016/S0006-3495(68)86471-9 4966691 PMC1367355

[B11] Fajardo-Cavazos P. Nicholson W. L. (1995). Molecular dissection of mutations in the *Bacillus subtilis* spore photoproduct lyase gene which affect repair of spore DNA damage caused by UV radiation. J. Bacteriol. 177, 4402–4409. 10.1128/jb.177.15.4402-4409.1995 7635825 PMC177190

[B12] Fajardo-Cavazos P. Rebeil R. Nicholson W. (2005). Essential cysteine residues in *Bacillus subtilis* spore photoproduct lyase identified by alanine scanning mutagenesis. Curr. Microbiol. 51, 331–335. 10.1007/s00284-005-0052-8 16163454

[B13] Fajardo-Cavazos P. Salazar C. Nicholson W. L. (1993). Molecular cloning and characterization of the *Bacillus subtilis* spore photoproduct lyase (*spl*) gene, which is involved in repair of UV radiation-induced DNA damage during spore germination. J. Bacteriol. 175, 1735–1744. 10.1128/jb.175.6.1735-1744.1993 8449881 PMC203968

[B14] Fish W. W. (1988). Rapid colorimetric micromethod for the quantitation of complexed iron in biological samples. Methods Enzymol. 158, 357–364. 10.1016/0076-6879(88)58067-9 3374387

[B15] Frey P. Magnusson O. T. (2003). S-adenosylmethionine: a wolf in sheep's clothing, or a rich man's adenosylcobalamin? Chem. Rev. 103, 2129–2148. 10.1021/cr020422m 12797826

[B16] Greenfield N. J. (2006). Using circular dichroism spectra to estimate protein secondary structure. Nat. Protoc. 1, 2876–2890. 10.1038/nprot.2006.202 17406547 PMC2728378

[B17] Kneuttinger A. C. Kashiwazaki G. Prill S. Heil K. Müller M. Carell T. (2014). Formation and direct repair of UV-induced dimeric DNA pyrimidine lesions. Photochem. Photobiol. 90, 1–14. 10.1111/php.12197 24354557

[B18] Kohen A. Limbach H.-H. (2006). Isotope Effects in Chemistry and Biology. Boca Raton, FL: Taylor and Francis.

[B19] Li J. Liu Z. Tan C. Guo X. Wang L. Sancar A.. (2010). Dynamics and mechanism of repair of ultraviolet-induced (6-4) photoproduct by photolyase. Nature 466, 887–890. 10.1038/nature09192 20657578 PMC3018752

[B20] Li L. (2012). Mechanistic studies of the radical SAM enzyme spore photoproduct lyase (SPL). Biochim. Biophys. Acta Proteins Proteomics 1824, 1264–1277. 10.1016/j.bbapap.2011.11.008 22197590 PMC3314140

[B21] Li L. Marsh E. N. G. (2006). Deuterium isotope effects in the unusual addition of toluene to fumarate catalyzed by benzylsuccinate synthase. Biochemistry 45, 13932–13938. 10.1021/bi061117o 17105211 PMC2519130

[B22] Lin G. Li L. (2010). Elucidation of spore-photoproduct formation by isotope labeling. Angew. Chem. Int. Ed. 49, 9926–9929. 10.1002/anie.201005228 21104967 PMC3132417

[B23] Liu S. Ji D. Cliffe L. Sabatini R. Wang Y. (2014). Quantitative mass spectrometry-based analysis of β-D-glucosyl-5-hydroxymethyluracil in genomic DNA of *Trypanosoma brucei*. J. Am. Soc. Mass Spectr. 25, 1763–1770. 10.1007/s13361-014-0960-6 25078157 PMC4163122

[B24] Liu Z. Tan C. Guo X. Kao Y.-T. Li J. Wang L.. (2011). Dynamics and mechanism of cyclobutane pyrimidine dimer repair by DNA photolyase. Proc. Natl. Acad. Sci. U.S.A. 108, 14831–14836. 10.1073/pnas.1110927108 21804035 PMC3169159

[B25] Mason J. M. Setlow P. (1986). Essential role of small, acid-soluble spore proteins in resistance of *Bacillus-subtilis* spores to UV-light. J. Bacteriol. 167, 174–178. 10.1128/jb.167.1.174-178.1986 3087950 PMC212857

[B26] Mehl R. A. Begley T. P. (1999). Mechanistic studies on the repair of a novel DNA photolesion: the spore photoproduct. Org. Lett. 1, 1065–1066. 10.1021/ol9908676 10825958

[B27] Moeller R. Douki T. Cadet J. Stackebrandt E. Nicholson W. L. Rettberg P.. (2007). UV-radiation-induced formation of DNA bipyrimidine photoproducts in *Bacillus subtilis* endospores and their repair during germination. Int. Microbiol. 10, 39–46. 10.2436/20.1501.01.6 17407059

[B28] Najbar L. V. Craik D. J. Wade J. D. McLeish M. J. (2000). Identification of initiation sites for T4 lysozyme folding using CD and NMR spectroscopy of peptide fragments. Biochemistry 39, 5911–5920. 10.1021/bi000070i 10801343

[B29] Najbar L. V. Craik D. J. Wade J. D. Salvatore D. McLeish M. J. (1997). Conformational analysis of LYS(11−36), a peptide derived from the β-sheet region of T4 lysozyme, in TFE and SDS. Biochemistry 36, 11525–11533. 10.1021/bi970730s 9298973

[B30] Northrop D. B. (1975). Steady-state analysis of kinetic isotope effects in enzymic reactions. Biochemistry 14, 2644–2651. 10.1021/bi00683a013 1148173

[B31] Sancar A. (2003). Structure and function of DNA photolyase and cryptochrome blue-light photoreceptors. Chem. Rev. 103, 2203–2238. 10.1021/cr0204348 12797829

[B32] Sancar A. (2008). Structure and function of photolyase and *in vivo* enzymology: 50th anniversary. J. Biol. Chem. 283, 32153–32157. 10.1074/jbc.R800052200 18682397 PMC2583285

[B33] Schramm V. L. (1998). Enzymatic transition states and transition state analog design. Annu. Rev. Biochem. 67, 693–720. 10.1146/annurev.biochem.67.1.693 9759501

[B34] Schramm V. L. (2007). Binding isotope effects: Boon and bane. Curr. Opion. Chem. Biol. 11, 529–536. 10.1016/j.cbpa.2007.07.013 17869163 PMC2066183

[B35] Setlow P. Li L. (2015). Photochemistry and photobiology of the spore photoproduct: a 50-year journey. Photochem. Photobiol. 91, 1263–1290. 10.1111/php.12506 26265564 PMC4631623

[B36] Sofia H. J. Chen G. Hetzler B. G. Reyes-Spindola J. F. Miller N. E. (2001). Radical SAM, a novel protein superfamily linking unresolved steps in familiar biosynthetic pathways with radical mechanisms: functional characterization using new analysis and information visualization methods. Nucleic Acids Res. 29, 1097–1106. 10.1093/nar/29.5.1097 11222759 PMC29726

[B37] Świderek K. Paneth P. (2013). Binding isotope effects. Chem. Rev. 113, 7851–7879. 10.1021/cr300515x 23848598

[B38] Wagner M. Steinbacher J. Kraus T. F. Michalakis S. Hackner B. Pfaffeneder T.. (2015). Age-dependent levels of 5-methyl-, 5-hydroxymethyl-, and 5-formylcytosine in human and mouse brain tissues. Angew. Chem. Int. Ed. 54, 12511–12514. 10.1002/anie.201502722 26137924 PMC4643189

[B39] Wang T.-C. V. Rupert C. S. (1977). Evidence for the monomerization of spore photoproduct to two thymines by the light-independent “spore repair” process in *Bacillus subtilis*. Photochem. Photobiol. 25, 123–127. 10.1111/j.1751-1097.1977.tb07432.x 403531

[B40] Yang L. Adhikari J. Gross M. L. Li L. (2017). Kinetic isotope effects and hydrogen/deuterium exchange reveal large conformational changes during the catalysis of the *Clostridium acetobutylicum* spore photoproduct lyase. Photochem. Photobiol. 93, 331–342. 10.1111/php.12697 27992649 PMC5315627

[B41] Yang L. Li L. (2013). The enzyme-mediated direct reversal of a dithymine photoproduct in germinating endospores. Int. J. Mol. Sci. 14, 13137–13153. 10.3390/ijms140713137 23799365 PMC3742179

[B42] Yang L. Li L. (2015). Spore photoproduct lyase: the known, the controversial, and the unknown. J. Biol. Chem. 290, 4003–4009. 10.1074/jbc.R114.573675 25477522 PMC4326811

[B43] Yang L. Lin G. Liu D. Dria K. J. Telser J. Li L. (2011). Probing the reaction mechanism of spore photoproduct lyase (SPL) via diastereoselectively labeled dinucleotide SP TpT substrates. J. Am. Chem. Soc. 133, 10434–10447. 10.1021/ja110196d 21671623 PMC3131481

[B44] Yang L. Lin G. Nelson R. S. Jian Y. Telser J. Li L. (2012). Mechanistic studies of the spore photoproduct lyase via a single cysteine mutation. Biochemistry 51, 7173–7188. 10.1021/bi3010945 22906093 PMC3448869

[B45] Yang L. Nelson R. S. Benjdia A. Lin G. Telser J. Stoll S.. (2013). A radical transfer pathway in spore photoproduct lyase. Biochemistry 52, 3041–3050. 10.1021/bi3016247 23607538 PMC3666868

