# Supplementary material for: Insights into the Activity Change of Spore Photoproduct Lyase Induced by Mutations at a Peripheral Glycine Residue
Source: Front Chem. 2017 Mar 28;5:14. doi: 10.3389/fchem.2017.00014 (PMC5368176; doi:10.3389/fchem.2017.00014)
Supplement: Supplementary file 1 [file Presentation1.PDF]

Supporting Information

For

Insights into the activity change of spore photoproduct lyase induced by mutations at a peripheral glycine residue

Linlin Yang,[1] and Lei Li[1,2]

[1]Department of Chemistry and Chemical Biology, Indiana University-Purdue University Indianapolis (IUPUI), 402 North Blackford Street, Indianapolis, Indiana, 46202, USA

[2]Department of Dermatology, Indiana University School of Medicine, Indianapolis, Indiana 46202, USA

lilei@iupui.edu

Table of Contents

	Page
ESI mass spectra for $SPL_{(Bs)}$ and its two glycine mutants.	S3
Deconvolved mass spectra for the SPL proteins	S4
CD spectra for the SPL proteins	S5

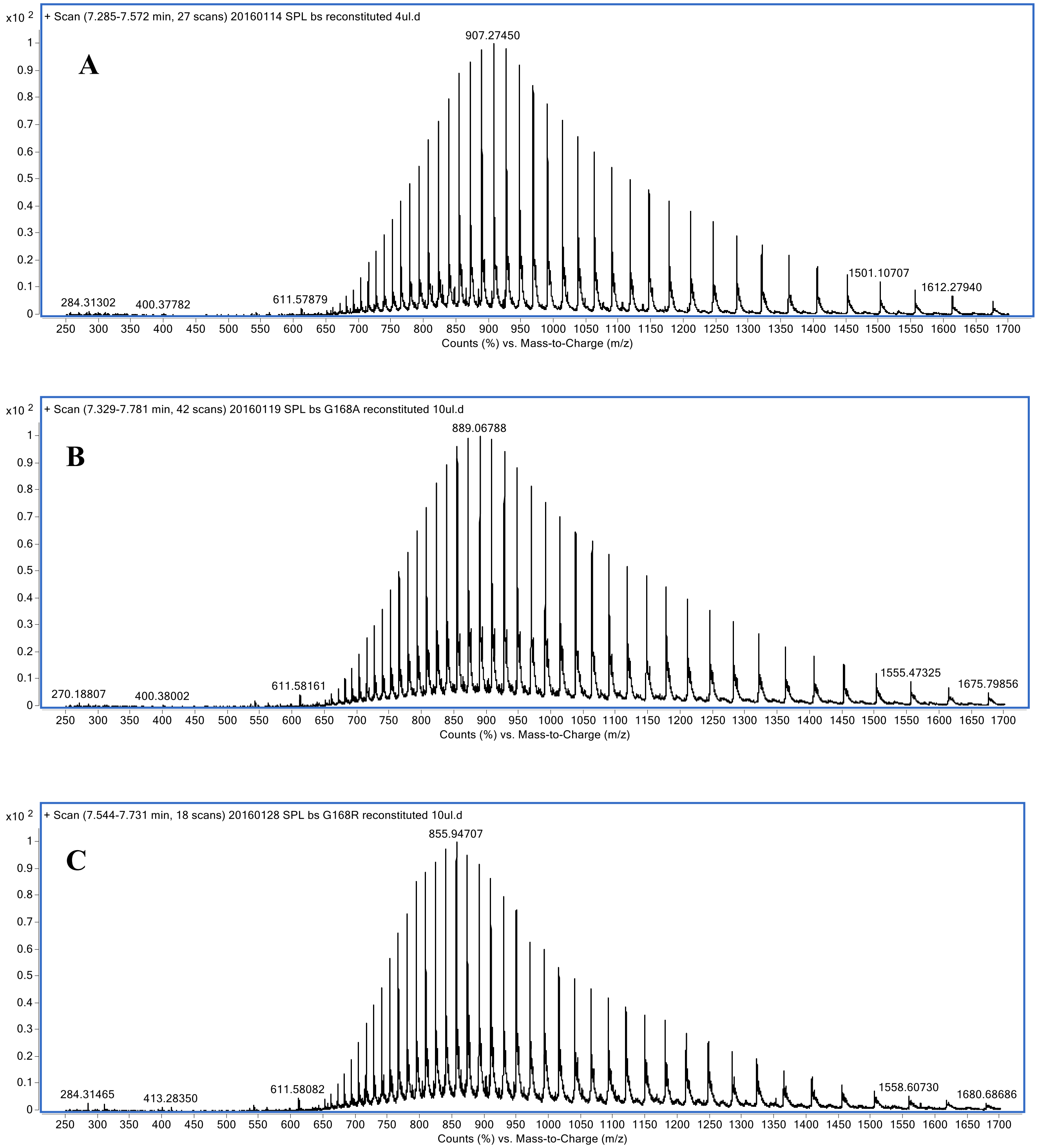

Figure S1. Electrospray mass spectrum of SPL$_{(Bs)}$ protein acquired under denaturing conditions. (A) WT SPL, (B) G168A mutant and (C) SPL G168R mutant. These SPL proteins contained the expression tag with the initiator methionine residue cleaved off.

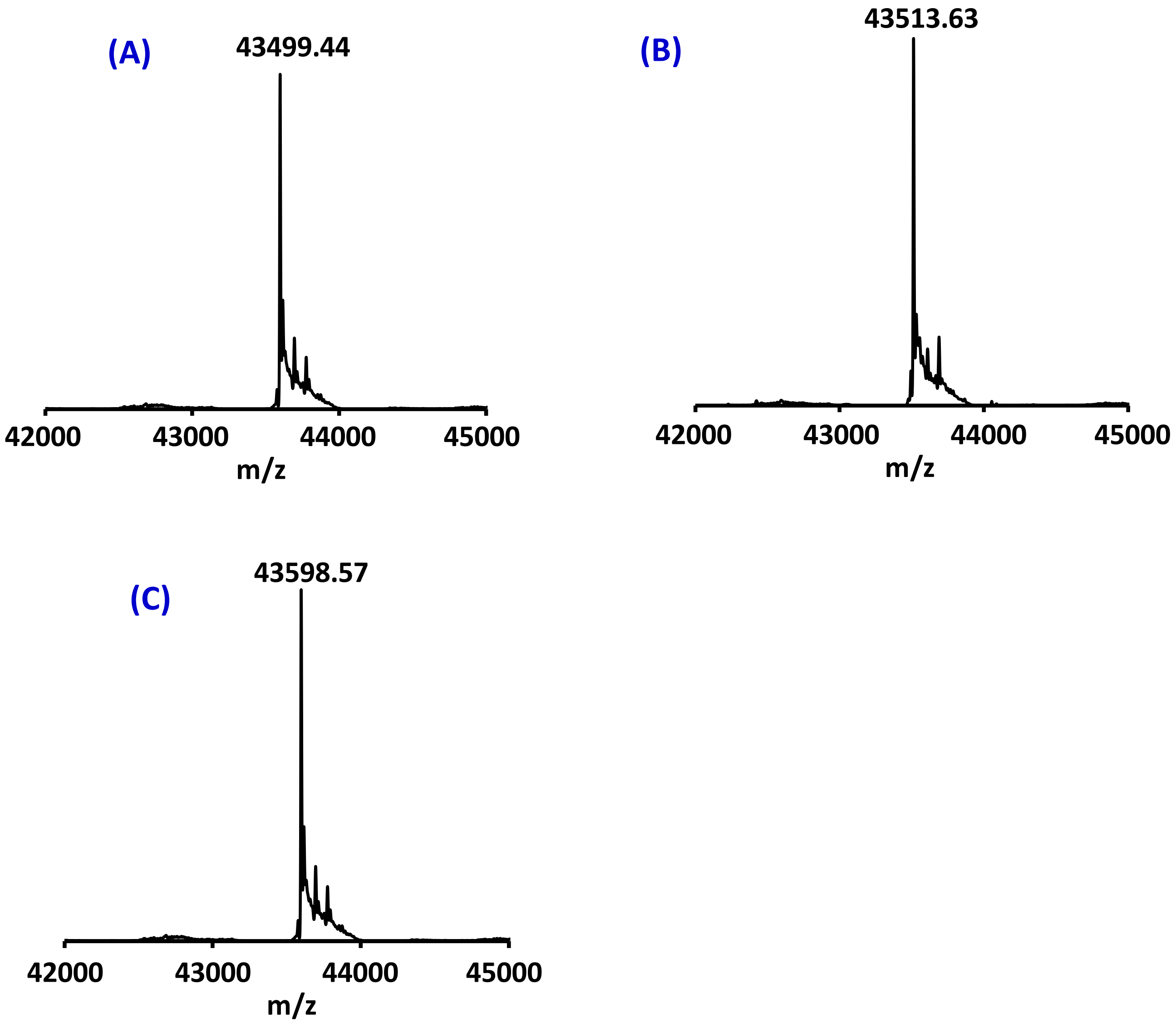

Figure S2. (A) Molecular weight determined for WT SPL (43499.44 Da) from the deconvolved mass spectrum, that agrees well with the calculated molecular weight (43500.36 Da). (B) Molecular weight determined for SPL G168A mutant (43513.63 Da) from the deconvolved mass spectrum, that agrees well with the calculated molecular weight (43514.36 Da). (C) Molecular weight determined for SPL G168R mutant (43598.57 Da) from the deconvolved mass spectrum, that agrees well with the calculated molecular weight (43599.36 Da). All three SPL proteins contain the expression tag minus the initiating methionine residue.

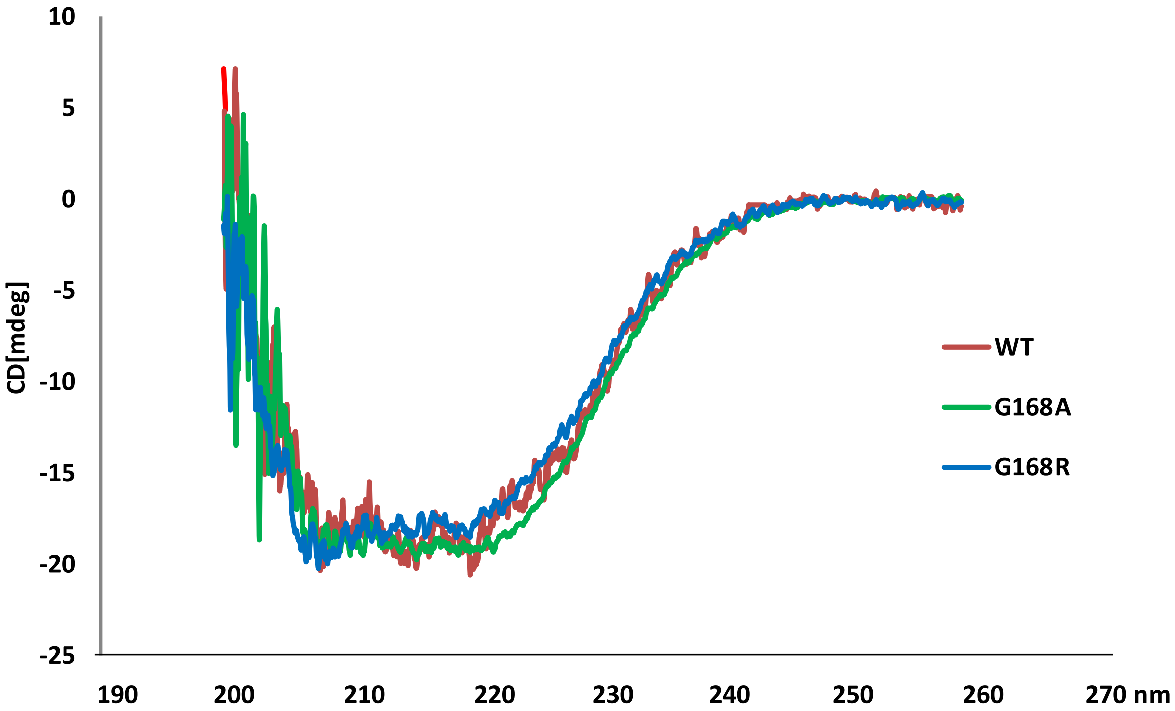

Figure S3. Far-UV CD spectra of WT SPL, SPL G168A and SPL G168R mutants at 288 K in a buffer containing 25 mM Tris, 250 mM NaCl and 10% glycerol at pH 8.
